# The SAMHD1-MX2 axis restricts HIV-1 infection at postviral DNA synthesis

**DOI:** 10.1128/mbio.01363-24

**Published:** 2024-06-18

**Authors:** Haoran Guo, Wanying Yang, Huili Li, Jiaxin Yang, Yuehan Huang, Yubin Tang, Shijin Wang, Fushun Ni, Weiming Yang, Xiao-Fang Yu, Wei Wei

**Affiliations:** 1 Institute of Virology and AIDS Research, First Hospital, Jilin University, Changchun, Jilin, China; 2 NIH/NIDCR, Bethesda, Maryland, USA; 3 Cancer Institute (Key Laboratory of Cancer Prevention and Intervention China National Ministry of Education), The Second Affiliated Hospital, Zhejiang University School of Medicine, Hangzhou, Zhejiang, China; 4 Cancer Center, Zhejiang University, Hangzhou, Zhejiang, China; 5 Key Laboratory of Organ Regeneration and Transplantation of Ministry of Education, Institute of Translational Medicine, First Hospital, Jilin University, Changchun, Jilin, China; University of Calgary, Calgary, Canada

**Keywords:** SAMHD1, MX2/MxB, HIV, antiviral immune defense, interferons

## Abstract

HIV-1 replication is tightly regulated in host cells, and various restriction factors have important roles in inhibiting viral replication. SAMHD1, a well-known restriction factor, suppresses HIV-1 replication by hydrolyzing intracellular dNTPs, thereby limiting the synthesis of viral cDNA in quiescent cells. In this study, we revealed an additional and distinct mechanism of SAMHD1 inhibition during the postviral cDNA synthesis stage. Using immunoprecipitation and mass spectrometry analysis, we demonstrated the interaction between SAMHD1 and MX2/MxB, an interferon-induced antiviral factor that inhibits HIV-1 cDNA nuclear import. The disruption of endogenous MX2 expression significantly weakened the ability of SAMHD1 to inhibit HIV-1. The crucial region within SAMHD1 that binds to MX2 has been identified. Notably, we found that SAMHD1 can act as a sensor that recognizes and binds to the incoming HIV-1 core, subsequently delivering it to the molecular trap formed by MX2, thereby blocking the nuclear entry of the HIV-1 core structure. SAMHD1 mutants unable to recognize the HIV-1 core showed a substantial decrease in antiviral activity. Certain mutations in HIV-1 capsids confer resistance to MX2 inhibition while maintaining susceptibility to suppression by the SAMHD1-MX2 axis. Overall, our study identifies an intriguing antiviral pattern wherein two distinct restriction factors, SAMHD1 and MX2, collaborate to establish an alternative mechanism deviating from their actions. These findings provide valuable insight into the complex immune defense networks against exogenous viral infections and have implications for the development of targeted anti-HIV therapeutics.

**IMPORTANCE:**

In contrast to most restriction factors that directly bind to viral components to exert their antiviral effects, SAMHD1, the only known deoxynucleotide triphosphate (dNTP) hydrolase in eukaryotes, indirectly inhibits viral replication in quiescent cells by reducing the pool of dNTP substrates available for viral cDNA synthesis. Our study provides a novel perspective on the antiviral functions of SAMHD1. In addition to its role in dNTP hydrolysis, SAMHD1 cooperates with MX2 to inhibit HIV-1 nuclear import. In this process, SAMHD1 acts as a sensor for incoming HIV-1 cores, detecting and binding to them, before subsequently delivering the complex to the molecular trap formed by MX2, thereby immobilizing the virus. This study not only reveals a new antiviral pathway for SAMHD1 but also identifies a unique collaboration and interaction between two distinct restriction factors, establishing a novel line of defense against HIV-1 infection, which challenges the traditional view of restriction factors acting independently. Overall, our findings further indicate the intricate complexity of the host immune defense network and provide potential targets for promoting host antiviral immune defense.

## INTRODUCTION

Sterile alpha-motif (SAM) domain and HD domain-containing protein 1 (SAMHD1), the only deoxynucleoside triphosphate triphosphohydrolase (dNTPase) in eukaryotes (1), have a pivotal role in the regulation of innate immune responses (2–4). Mutations in SAMHD1 have been associated with autoimmune diseases such as Aicardi–Goutières syndrome (AGS), which are characterized by aberrant immune activation (2). SAMHD1 was also shown to have potent cellular restriction activity against retroviruses in myeloid cells and resting CD4^+^ T cells (5–8) and to suppress endogenous retrotransposons (9–11). The dNTPase activity of SAMHD1 is activated by dGTP/GTP when it forms a homotetramer, leading to intracellular dNTP depletion and subsequent retroviral restriction specifically in myeloid cells and resting CD4^+^ T cells (12). However, the regulatory mechanisms underlying the antiviral function of SAMHD1 have not been fully elucidated.

Human myxovirus resistance protein 2 (MX2/MxB) was recently identified as a key mediator of interferon (IFN)-induced inhibition of HIV-1 replication (13–15). In humans, the presence of two myxovirus resistance genes, *mx1* and *mx2*, has been attributed to gene duplication, which resulted in the IFN-inducible dynamin-like GTPases, MX1/MxA, and MX2/MxB. Human MX1 was shown to suppress a broad spectrum of DNA and RNA viruses, including influenza A virus (IAV) and hepatitis B virus (HBV) (16, 17). MX2 was also found to have inhibitory effects on HIV-1, herpesviruses, and HBV (18, 19). MX2 has been shown to exert a suppressive effect on HIV-1 prior to the nuclear import of viral preintegration complexes, which occurs after viral DNA synthesis through reverse transcription (20–23). Furthermore, point mutations in the HIV-1 capsid (CAp24) protein have been shown to confer resistance to MX2, leading to the accumulation of MX2-resistant HIV-1 capsid variants during the expansion of the HIV-1 epidemic in human populations (24–26). The existence of additional unknown cofactors or alternative antiviral pathways involved in the anti-HIV-1 activity of MX2 remains unclear.

In the present study, we identified an additional and distinct mechanism of inhibition mediated by SAMHD1 during the postviral cDNA synthesis stage that is dependent on MX2. SAMHD1 functions as a sensor for HIV-1 cores, recognizing and binding to them before delivering them to the molecular trap formed by MX2, effectively blocking HIV-1 nuclear entry. Our study revealed a collaborative interaction between two separate restriction factors, SAMHD1 and MX2, which collectively suppress HIV-1 replication beyond their actions.

## RESULTS

### SAMHD1 exerts a potent alternative inhibitory effect on HIV-1 replication after viral reverse transcription

**Fig 1:**
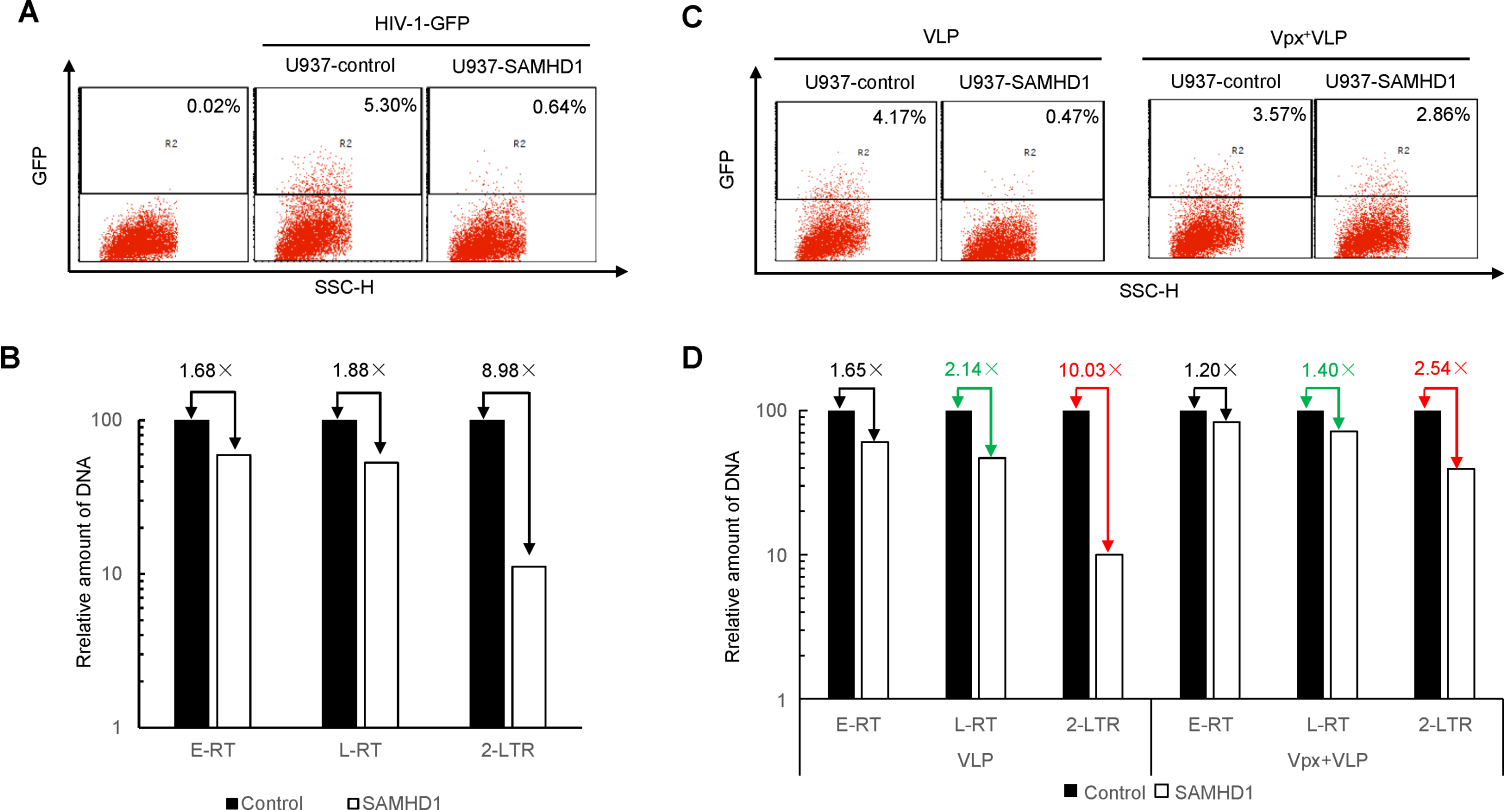
Vpx impairs SAMHD1-mediated HIV-1 inhibition post-reverse transcription. (**A**) HIV-1 restriction by SAMHD1 in differentiated U937 cells. SAMHD1 was transduced into U937 cells using lentiviral vectors as described in Materials and Methods to generate U937-SAMHD1 cells. U937 or U937-SAMHD1 cells were treated with PMA (100 ng/mL for 24 h). The two groups of treated cells were infected with equivalent amounts of the HIV-1-GFP virus. The percentage of GFP-positive cells was determined by flow cytometry 2 days after infection. (**B**) SAMHD1 inhibits the nuclear accumulation of HIV-1 cDNA. Quantitative PCR analysis of reverse-transcript and 2-LTR circle viral cDNA in the control or SAMHD1-expressing U937 cells. (**C**) Vpx+ VLP increased HIV-1 infection in the presence of SAMHD1 in differentiated U937 cells. U937 or U937-SAMHD1 cells were pretreated with PMA (100 ng/mL) for 24 h and then treated with Vpx+ VLP or Vpx− VLP for 2 h. The various treated U937 cells were infected with equivalent amounts of HIV-1-GFP virus. (**D**) Vpx+ VLP increased HIV-1 2-LTR cDNA accumulation in U937/SAMHD1 cells.

The dNTPase activity of SAMHD1 restricts HIV-1 reverse transcription in myeloid cells (27). Interestingly, we discovered that SAMHD1 can have a profound antiviral effect at the post-reverse transcription stage. Transduction of differentiated U937 macrophages with SAMHD1 resulted in approximately 90% inhibition of HIV-1 (Fig. 1A). The 2-long terminal repeat (2-LTR) cDNA circle is formed through the ligation of nuclear cDNA ends by the nonhomologous DNA end-joining system of the host cell (28, 29). The accumulation of viral 2-LTR cDNA, which is a marker for nuclear accumulation of viral DNA, was significantly reduced in differentiated SAMHD1-positive U937 macrophages compared to SAMHD1-negative U937 cells, showing an 8.98-fold decrease (Fig. 1B). The magnitude of SAMHD1 inhibition of the viral 2-LTR product (Fig. 1B) matched the SAMHD1-mediated suppression of viral infectivity (Fig. 1A). SAMHD1-mediated inhibition of HIV-1 2-LTR cDNA accumulation was neutralized by pretreatment with Vpx+ VLP (Fig. 1C and D), which induces the degradation of SAMHD1 (Fig. S1).

### SAMHD1 interacts with MX2

**Fig 2:**
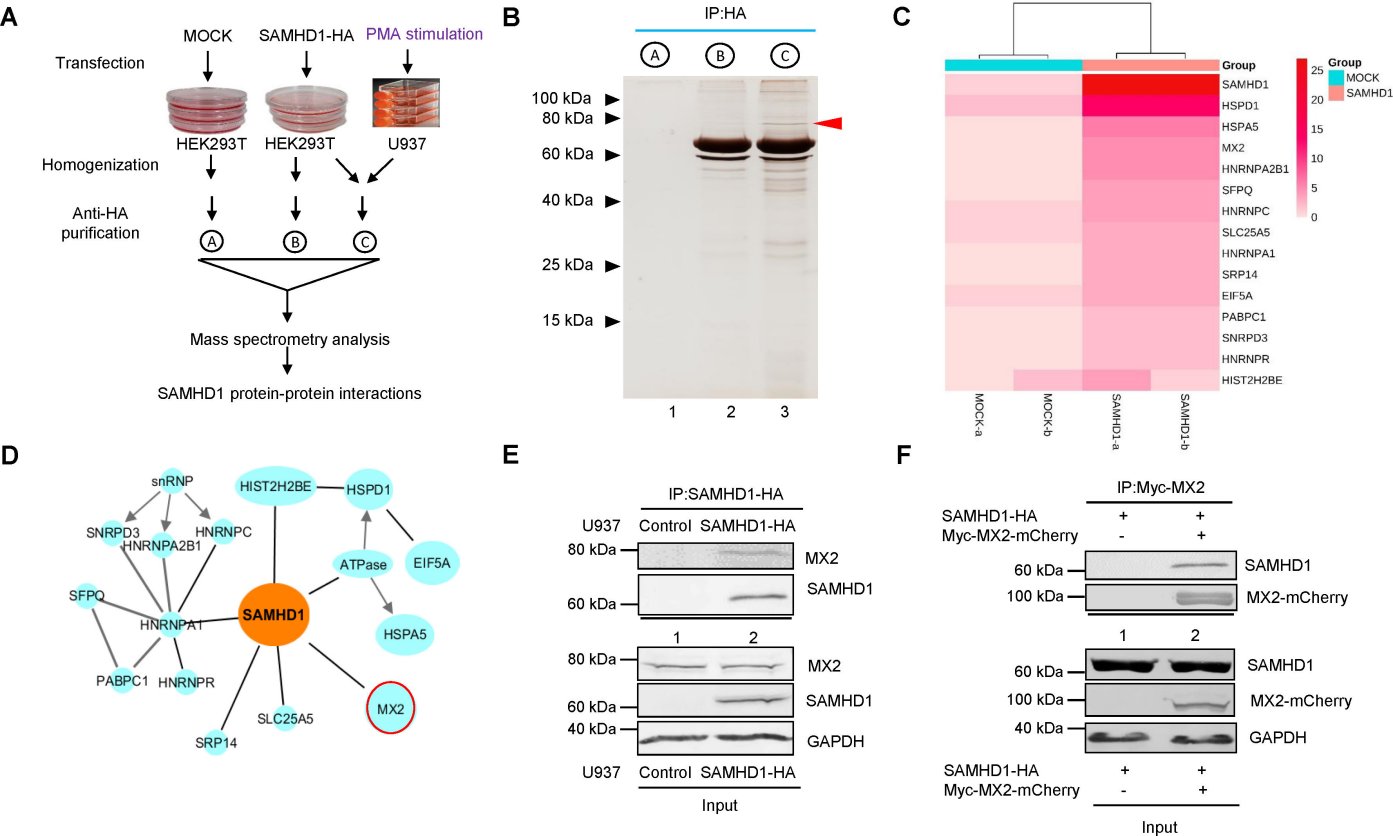
Characterization of the interaction between SAMHD1 and MX2. (**A**) Schematic of the screens used to identify candidate proteins involved in SAMHD1-dependent anti-HIV-1 activity. (**B**) Cell lysates from the PMA-treated U937 cells and HEK293T cells transfected with SAMHD1-HA or the control vector were mixed and then immunoprecipitated (IP) with an anti-HA antibody conjugated to agarose beads, followed by SDS-PAGE and silver staining. (**C**) Heatmap representation of candidate proteins identified as SAMHD1 binding partners in differentiated U937 cells. (**D**) Network representation of candidate proteins identified as SAMHD1 binding partners in differentiated U937 cells. (**E**) Co-IP and immunoblotting confirm the interaction of endogenous MX2 with SAMHD1. (**F**) Co-IP of SAMHD1-HA with Myc-MX2-mCherry from transfected HEK293T cell samples.

SAMHD1 mediates this antiviral activity in U937 myeloid cells but not in other cell types such as HEK293T cells, suggesting that specific cellular cofactors are involved in the antiviral activity of SAMHD1 (Fig. S2). We therefore performed a proteomic analysis of SAMHD1 cofactors in HEK293T cells and differentiated U937 cells (Fig. 2A). Cellular proteins associated with HA-tagged SAMHD1 in HEK293T cells and differentiated U937 cells were immunoprecipitated and analyzed by mass spectrometry (Fig. 2B). Several proteins were found to uniquely associate with SAMHD1 (Fig. 2C and D ). One of these proteins has a predicted molecular mass of approximately 80 kDa and was confirmed to be MX2 by immunoblotting with an MX2-specific antibody . The interaction between SAMHD1 and MX2 was further confirmed using epitope-tagged proteins (Fig. 2E and F; Fig. S3). This SAMHD1-MX2 interaction is particularly interesting because MX2 has been reported to be an IFN-induced inhibitor of HIV-1, reducing the accumulation of viral 2-LTR cDNA, consistent with the SAMHD1-mediated antiviral phenomenon observed above (Fig. 1).

### The critical regions in SAMHD1 for MX2 binding

**Fig 3:**
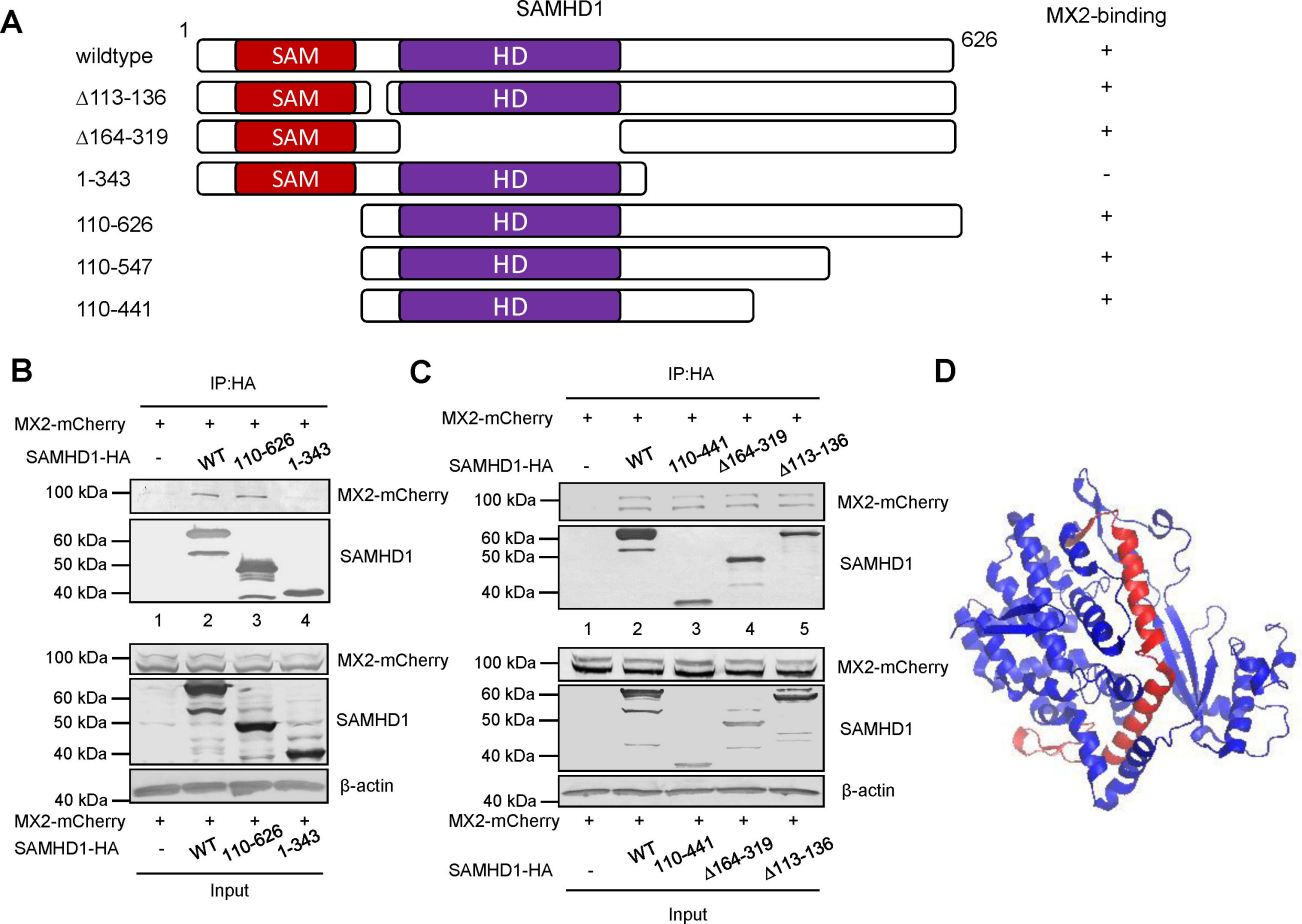
The critical region of SAMHD1 is important for MX2 binding. (**A**) Schematic representation of constructed full-length and truncated variants of SAMHD1. The SAMHD1 mutant shows an impaired interaction with MX2, marked as –. (**B and C**) The indicated cell lysates were prepared 48 h after transfection and immunoprecipitated with an anti-HA affinity matrix (Roche). The interaction of MX2 with WT or mutated SAMHD1-HA molecules was detected by immunoblotting with an anti-HA antibody to detect SAMHD1-HA and an anti-MX2 antibody to detect mCherry-MX2. The input cell lysates were detected by immunoblotting for the indicated proteins. (**D**) The SAMHD1 region that is important for the interaction with MX2 is marked in red in the SAMHD1 crystal structure.

**Fig 4:**
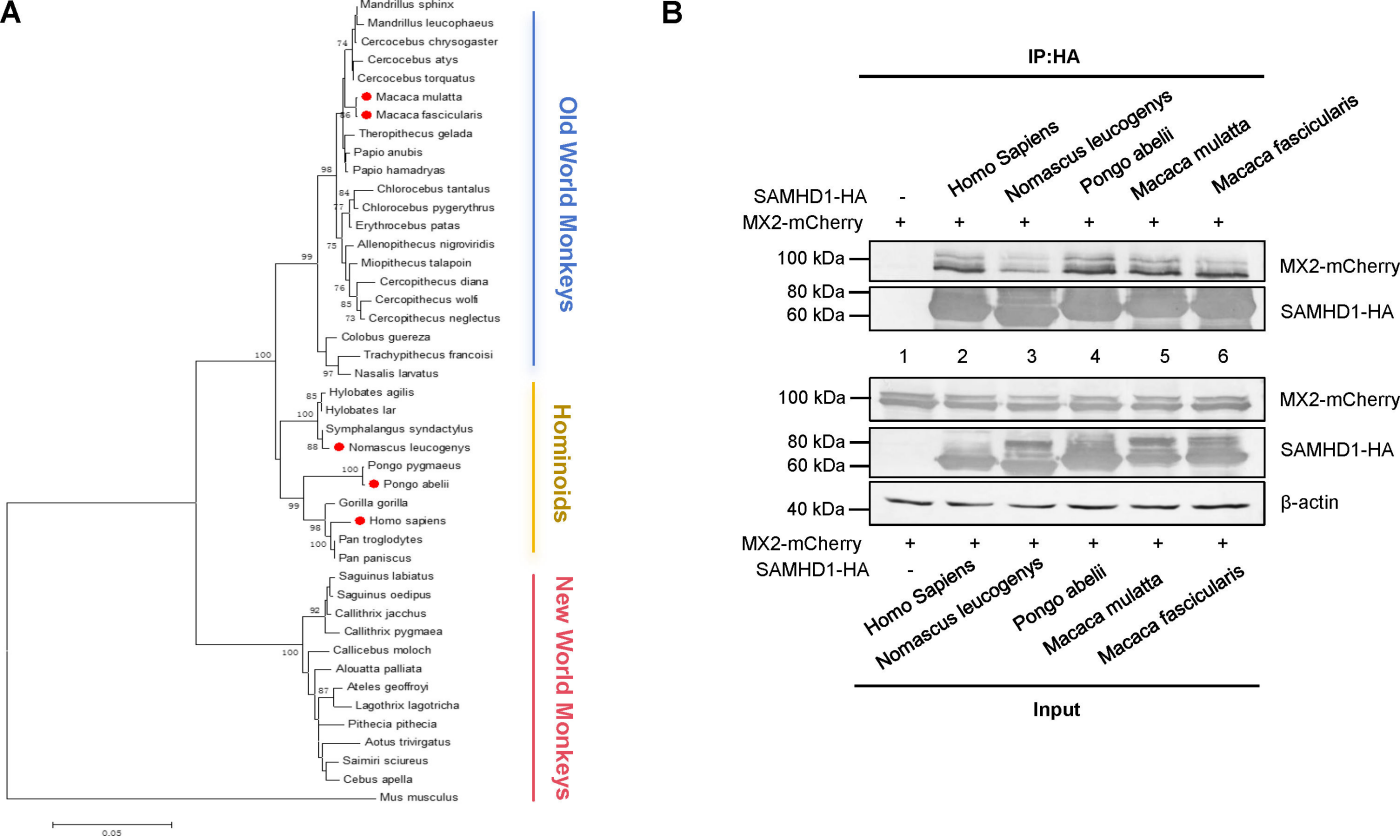
Primate SAMHD1 proteins interact with human MX2. (**A**) Phylogenetic analysis of primate SAMHD1. The coding sequences of primate SAMHD1 molecules and Mus musculus SAMHD1 were retrieved from GeneBank. Phylogenetic analysis was conducted with MEGA4 software using the neighbor-joining method, with 1,000 replications and the Kimura 2-parameter model. Bootstrap values >70% are shown. The five SAMHD1 constructs we used in this study are labeled with red dots. (**B**) HEK293T cells were cotransfected with a mCherry-fused MX2 expression vector plus a control vector, human SAMHD1-HA, or one of the indicated primate SAMHD1s. Cell lysates were prepared 48 h after transfection and immunoprecipitated with an anti-HA affinity matrix (Roche). The interaction of MX2 with primate SAMHD1 molecules was detected by immunoblotting with the indicated antibodies.

We then identified the critical region in SAMHD1 that is required for the MX2 interaction (Fig. 3A). A region (aa 343 to 441) in the HD domain of SAMHD1 was found to be important for MX2 binding (Fig. 3B and C). This region was reported to be critical for the anti-HIV-1 function of SAMHD1 (30). Furthermore, amino acids in this region are exposed on the surface of SAMHD1, as indicated by its crystal structure (Fig. 3D). MX2 also interacted with SAMHD1 molecules from diverse primates, consistent with the conserved nature of this region in primate SAMHD1 molecules (Fig. 4A and B).

### SAMHD1 cooperates with MX2 to inhibit HIV-1 infectivity

**Fig 5:**
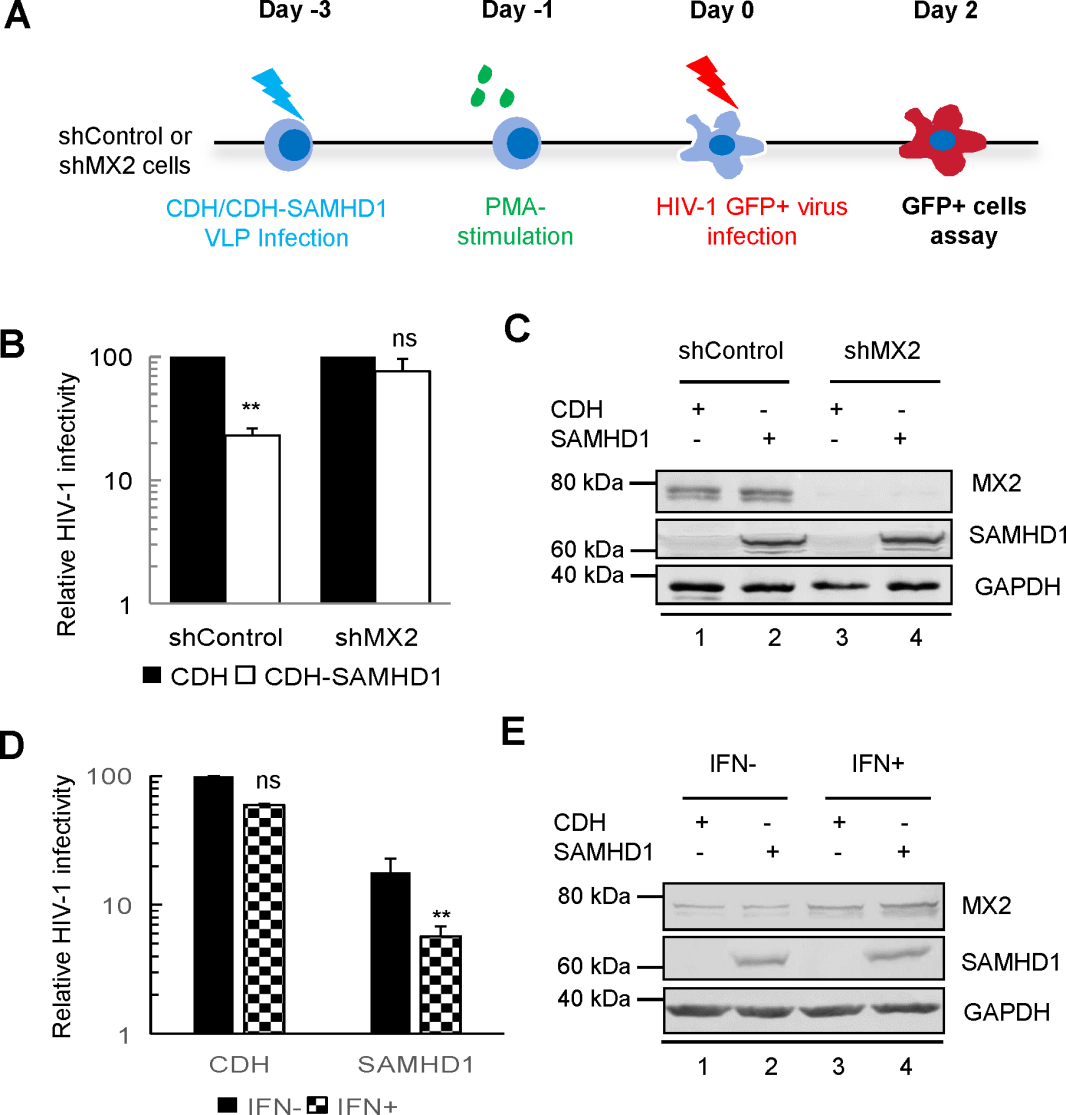
MX2 and SAMHD1 collaborate to inhibit HIV-1 infectivity. (**A**) Flow chart depicting the HIV-1 GFP reporter infectivity analysis performed in the study. (**B**) U937 cells expressing control shRNA or shRNA targeting MX2 were infected with the CDH-SAMHD1 or CDH virus for 48 h. The cells were stimulated with PMA for another 24 h, and two groups of treated cells were infected with equivalent amounts of HIV-1-GFP. The percentage of GFP-positive cells was determined by flow cytometry 2 days after infection. The mean relative infection efficiencies from three independent experiments are shown. (**C**) Immunoblot analysis of parallel samples from panel B. (**D**) For determination of whether SAMHD1 affects IFN-induced anti-HIV-1 activity, U937 or U937 cells expressing SAMHD1 were treated with IFNα for 18 h. Cells were then infected with the HIV-1-GFP virus, and GFP-positive cells were determined by flow cytometry 48 h after infection. (**E**) Immunoblot analysis of parallel samples from panel D.

To determine whether MX2, an IFN-induced host anti-HIV-1 factor that blocks the nuclear accumulation of viral cDNA, is involved in the anti-HIV-1 activity of SAMHD1, we silenced MX2 in U937 cells using an MX2-specific shRNA (Fig. 5A). Silencing MX2 did not affect HIV-1 infectivity in U937 cells compared to that in the control (unsilenced) U937 cells. However, in the MX2-silenced U937 cells, the antiviral activity of SAMHD1 was significantly compromised compared to that in the MX2-normal U937 cells (Fig. 5B). SAMHD1 inhibited HIV-1 infection by 80% in the control U937 cells (Fig. 5B, compare bars 1 and 2). In the MX2-silenced U937 cells, the antiviral activity of SAMHD1 was <10% (Fig. 5B, compare bars 3 and 4). Silencing MX2 did not, however, alter the expression of SAMHD1 (Fig. 5C).

We then investigated whether increasing MX2 expression by IFN treatment increased the antiviral activity of SAMHD1. In SAMHD1-null U937 cells, treatment with IFNα had only a minor effect (1.6-fold reduction) on HIV-1 infection (Fig. 5D). By contrast, in SAMHD1-positive U937 cells, interferon-α (IFNα) treatment resulted in a 3.1-fold inhibition of HIV-1 infection. IFNα treatment also resulted in a twofold to threefold increase in MX2 expression in these cells (Fig. 5E). Thus, increased MX2 expression after IFNα treatment had a limited effect on HIV-1 infection in the U937 cells lacking SAMHD1, but in the SAMHD1-expressing U937 cells, IFNα treatment both increased MX2 expression and promoted HIV-1 suppression.

**Fig 6:**
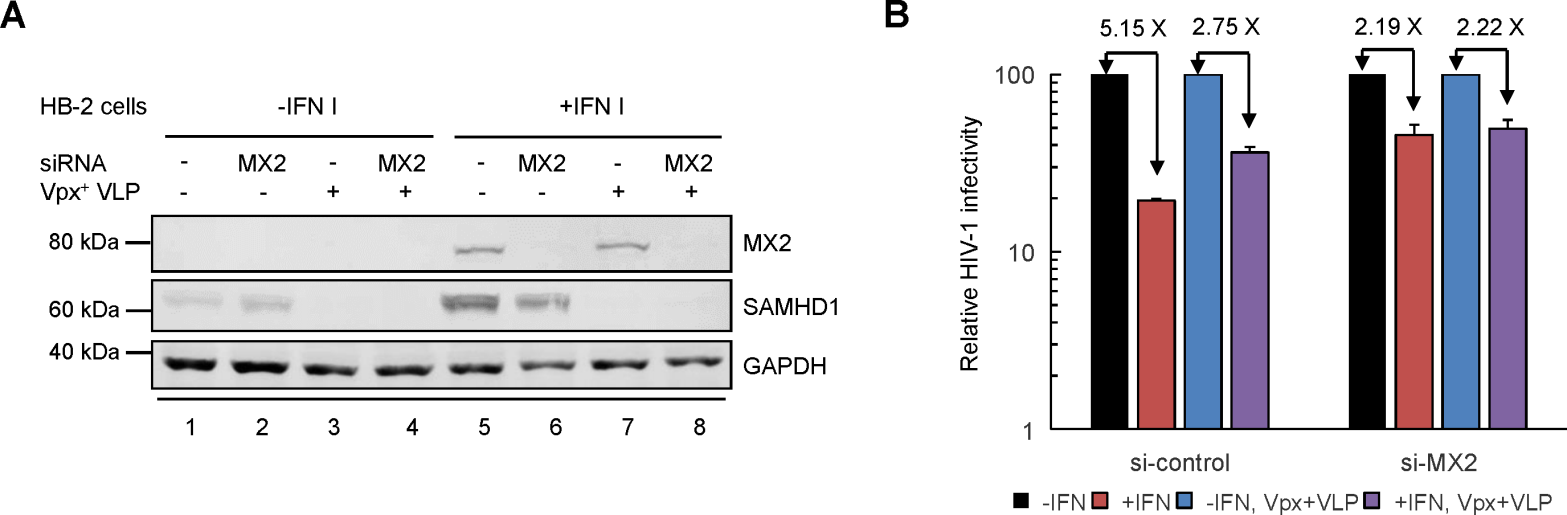
Knockdown of MX2 expression impairs Vpx-induced increases in HIV-1 infection in the IFN-treated human dendritic cell line HB-2. HB-2 cells transfected with control siRNA or siRNA targeting MX2 were cultured with or without IFN-α (500  U/mL) for 18 h. The treated cells were then infected with equivalent amounts of the HIV-1-GFP virus. (**A**) Protein expression of MX2 and SAMHD1 in these cells was assessed by immunoblotting. (**B**)The percentage of HIV-1-infected GFP-positive cells was determined by flow cytometry 2 days after infection.

In addition, in the human dendritic cell-derived cell line HB-2, IFNα treatment resulted in MX2 upregulation (Fig. 6A) and ~80% HIV-1 inhibition (Fig. 6B, compare bars 1 and 2). Depletion of SAMHD1 by Vpx+ VLP attenuated IFNα-induced anti-HIV-1 activity (Fig. 6B, compare bars 2 and 4). By contrast, Vpx+ VLP treatment did not affect IFNα-induced anti-HIV-1 activity in the MX2-silenced HB-2 cells (Fig. 6B, bars 5–8). Thus, SAMHD1 is involved in IFNα-induced HIV-1 suppression only in the presence of MX2. Collectively, these data indicate that SAMHD1 mediates its anti-HIV-1 activity by engaging MX2.

### Distinct patterns of MX2-dependent inhibition of HIV-1 infection

**Fig 7:**
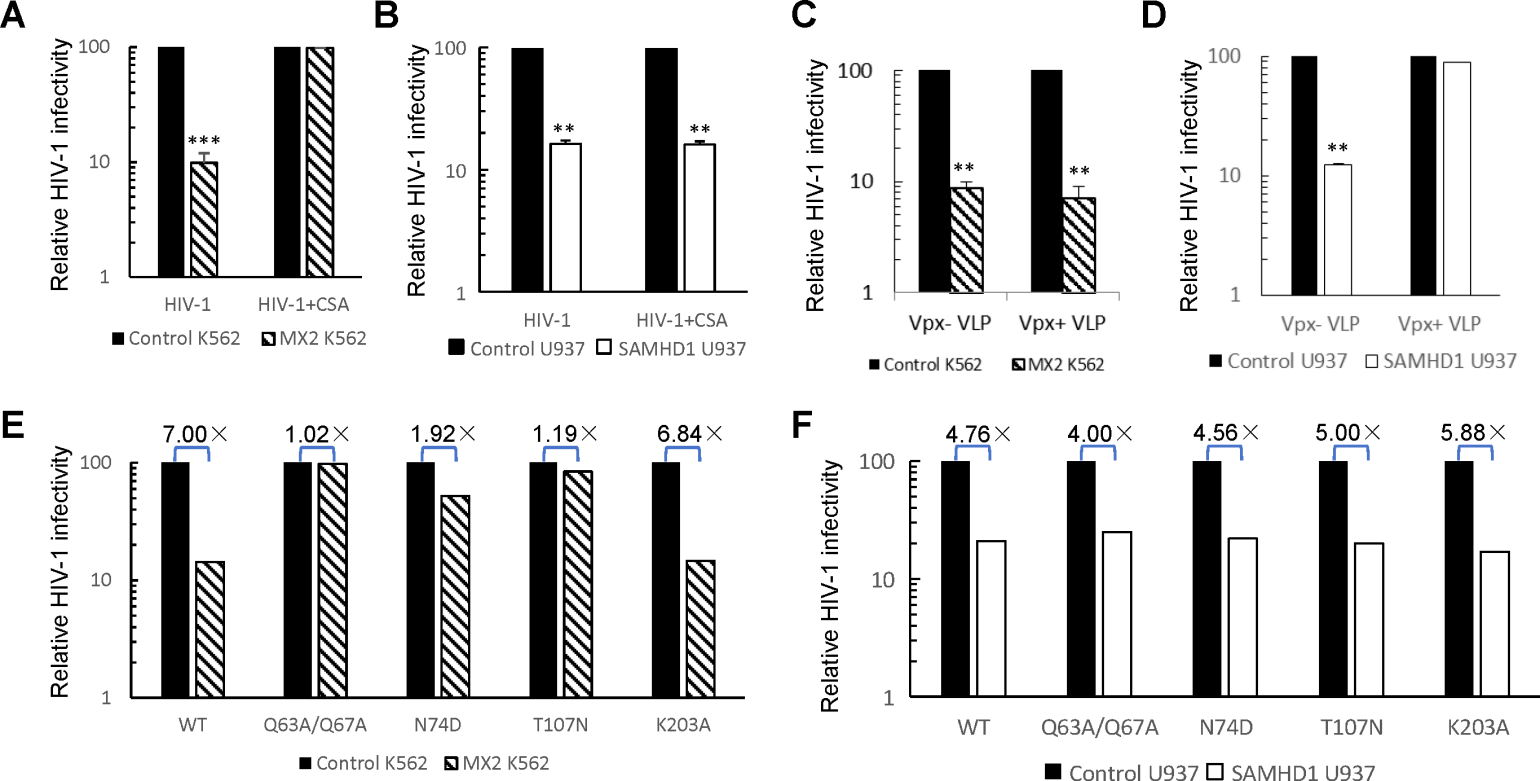
Distinct patterns of MX2-dependent inhibition of HIV-1 infection. (**A**) Treatment with CsA abolished MX2-mediated antiviral activity in K562 cells. K562 or MX2-transduced K562 cells (untreated or treated with CsA) were infected with equal amounts of GFP-expressing HIV-1 viruses. The number of GFP^+^ cells was determined 2 days after infection by flow cytometry. (**B**) Treatment with CsA did not affect SAMHD1/MX2-mediated antiviral activity in U937 cells. (**C**) Treatment with Vpx+ VLP did not affect MX2-mediated antiviral activity in K562 cells. (**D**) Treatment with Vpx+ VLP abolished SAMHD1/MX2-mediated antiviral activity in U937 cells. (**E**) Identification of CA mutants resistant to MX2-mediated antiviral activity in K562 cells. (**F**) CA mutants resistant to MX2-mediated antiviral activity in K562 cells were still sensitive to SAMHD1/MX2-mediated antiviral activity in U937 cells.

The anti-HIV-1 activity of MX2 depends on the expression of cyclophilin A (CypA) and possibly other host factors. Consistent with previous reports, the antiviral activity of MX2 was essentially neutralized by treatment with the CypA inhibitor cyclosporine (CsA) in K562 cells (Fig. 7A). CsA did not affect HIV-1 infectivity in K562 cells lacking MX2 (Fig. 7A). However, MX2-dependent SAMHD1-mediated anti-HIV-1 activity in U937 cells was not affected by CsA treatment (Fig. 7B). Vpx+ VLP did not affect MX2-mediated anti-HIV-1 activity in K562 cells (Fig. 7C). By contrast, SAMHD1-dependent MX2-mediated antiviral activity was inhibited by Vpx+ VLP treatment in U937 cells (Fig. 7D). Thus, although the antiviral activity of SAMHD1 depends on the presence of MX2, this activity is distinct from the CypA-dependent MX2-mediated anti-HIV-1 activity.

The MX2-mediated antiviral activity in K562 cells was also attenuated by CA mutation, specifically a CA mutant (N74D) defective in CPSF6 binding (Fig. 7E). However, HIV-1 (CA-N74D) was still sensitive to SAMHD1-mediated inhibition in U937 cells (Fig. 7F). We identified several novel CA mutants that showed increased resistance to MX2-mediated inhibition in K562 cells (Fig. 7E). All of these mutants were still suppressed by SAMHD1/MX2 in U937 cells (Fig. 7F). Thus, SAMHD1 and CypA/CPSF6 both engage MX2 and have nonoverlapping anti-HIV-1 activities.

### SAMHD1 is an HIV-1 capsid-targeting factor

**Fig 8:**
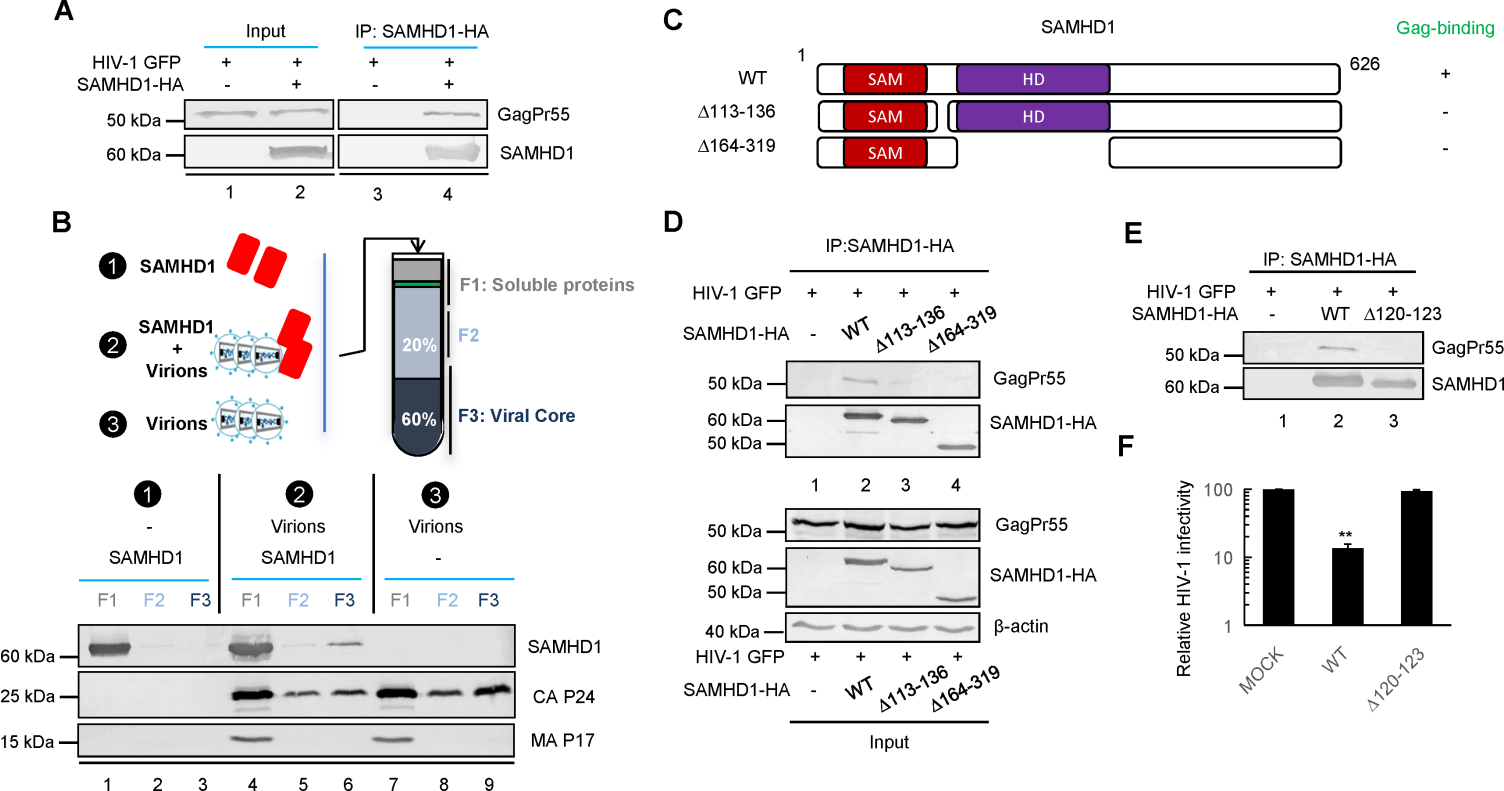
SAMHD1 is an HIV-1 capsid-targeting factor. (**A**) SAMHD1 interaction with HIV-1 Gag molecules. HEK293T cells were transfected HIV-1 (NL4-3) with or without the SAMHD1-HA expression vector. Co-IP was carried out 48 h later using an anti-HA affinity matrix (Roche). Protein samples were analyzed by immunoblotting with an anti-p24 monoclonal antibody to detect GagPr55 and an anti-HA antibody to detect SAMHD1-HA. (**B**) Schematic representation of HIV-1 core purification in the presence or absence of SAMHD1 (upper). Association of SAMHD1 with HIV-1 cores (bottom). The core fraction (F3) contains CAp24 but is largely devoid of MAp17. (**C**) Schematic representation of constructed full-length SAMHD1 and two deletion variants of SAMHD1. SAMHD1 mutants that impair the interaction with Gag are marked with a –. (**D**) Interaction of GagPr55 with WT or mutated SAMHD1. (**E**) The SAMHD1 linker region (120–123 aa) that is important for the interaction with GagPr55. (**F**) SAMHD1 Δ120–123 loses the capacity to suppress HIV-1 infection.

**Fig 9:**
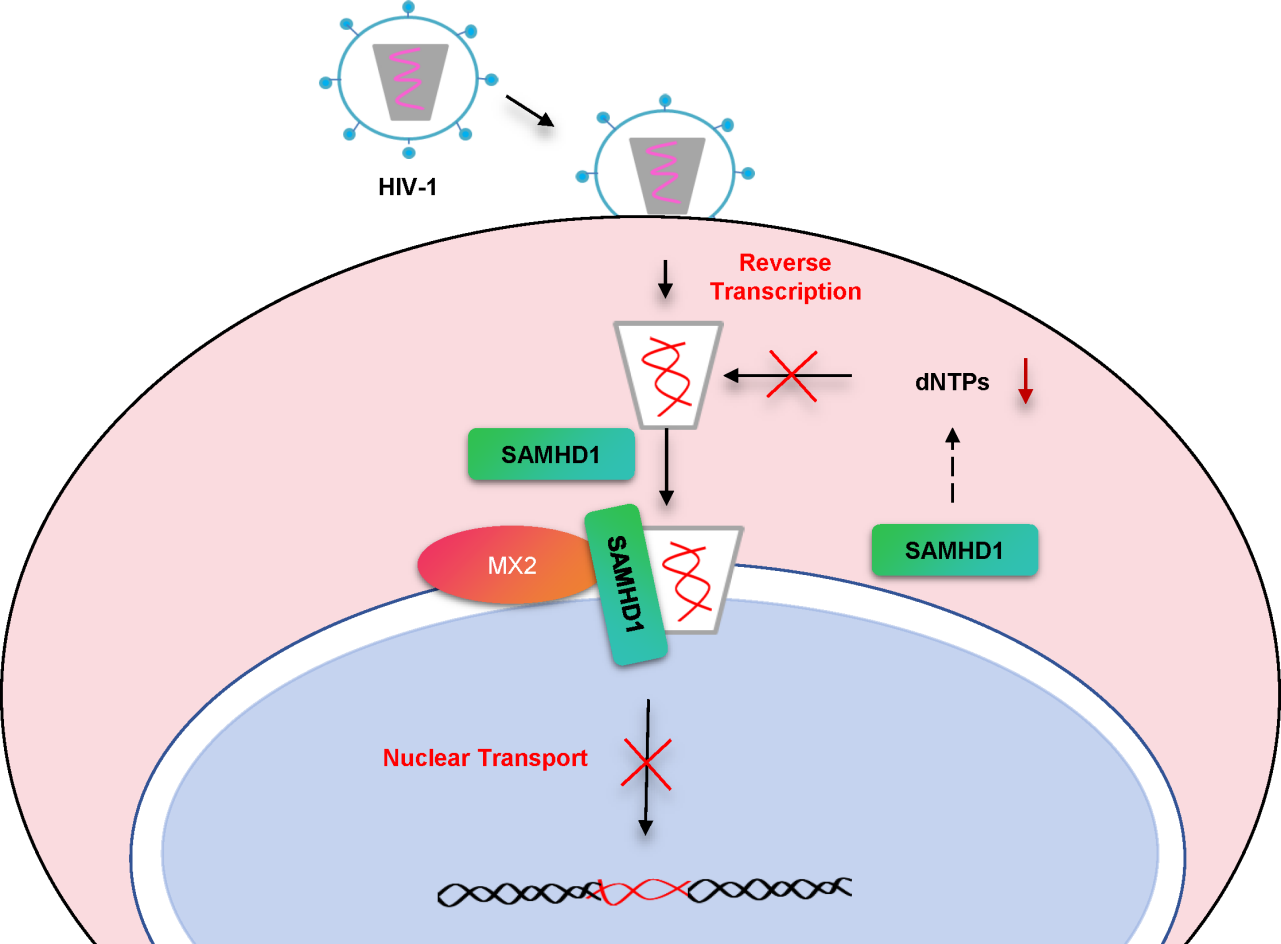
Model of two distinct pathways of SAMHD1-mediated HIV-1 restriction that are inactivated by Vpx. SAMHD1 decreases the cellular dNTP pool to block HIV-1 reverse transcription and interacts with IFN-induced MX2 to sense the incoming viral core to inhibit viral cDNA accumulation in the nucleus.

MX2 inhibition of HIV-1 requires CypA binding to HIV-1 CAp24. In the absence of functional CypA (CsA-treated U937 cells), SAMHD1/MX2 still inhibited HIV-1 infection. This resistance raises the question of whether SAMHD1 recognition of HIV-1 Gag proteins triggers the antiviral function of MX2. The SAMHD1-HIV-1 Gag interaction was first evaluated by coimmunoprecipitation experiments (Co-IP). We indeed identified a specific interaction between SAMHD1 and GagPr55 of HIV-1 when these two proteins were coexpressed in HEK293T cells (Fig. 8A). The ability of SAMHD1 to bind to Gag suggests that intracellular SAMHD1 recognizes the incoming HIV-1 core after virus‒cell fusion. We next investigated whether SAMHD1 can associate with the viral core (Fig. 8B) using a well-established viral core purification strategy. Copurification of SAMHD1 with the HIV-1 core was indeed observed (Fig. 8B). In the absence of HIV-1, SAMHD1 was not found in the same fraction, indicating a specific interaction between SAMHD1 and the HIV-1 core protein. Whether SAMHD1 requires other cofactors to bind the HIV-1 capsid remains to be determined. The SAMHD1-HIV-1 Gag interaction was disrupted by an internal deletion in the linker region or the HD domain of SAMHD1 (Fig. 8C and D). Furthermore, a SAMHD1 variant (∆120–123) from Aicardi-Goutières syndrome (AGS) patients was defective in HIV-1 Gag binding (Fig. 8E). This SAMHD1 mutant lost the ability to inhibit HIV-1 infection in differentiated U937 cells (Fig. 8F). These data demonstrate that recognition of HIV-1 Gag molecules by SAMHD1 is important for its antiviral function (Fig. 9).

## DISCUSSION

After an extensive period of research and exploration, SAMHD1 was ultimately identified as a natural antiviral agent. Initially, we and others reported the presence of Vpx and confirmed its critical role in viral infection in macrophages (31–34). Subsequent studies have indicated that Vpx facilitates viral infection in macrophages by recruiting the CRL4(DCAF1) ubiquitin ligase complex and inducing the degradation of an unidentified antiviral factor (35, 36). The identification of Vpx’s host-binding proteins through mass spectrometry successfully identified SAMHD1 as the primary target for Vpx-mediated degradation (1, 5). SAMHD1 effectively inhibits HIV/SIV replication in nondividing cells, such as macrophages, dendritic cells, and resting CD4+ T cells (1, 5, 7). The Vpx proteins encoded by HIV-2 and certain SIV strains induce SAMHD1 degradation via the formation of the Vpx-CRL4(DCAF1) complex, thereby disrupting the host cellular defense mechanism dependent on SAMHD1 (37–39). Notably, despite HIV-1 being the primary pathogen responsible for global AIDS, its inability to encode the Vpx protein hampers its capacity to overcome the antiviral defense by SAMHD1 in macrophages. Hence, a comprehensive understanding of the antiviral mechanisms of SAMHD1 not only helps elucidate molecular strategies underlying host innate immune defense but also provides insights and design strategies for targeted increases in SAMHD1 activity against HIV-1. In this study, we identify a novel mechanism by which SAMHD1 exerts its inhibitory effect on HIV-1 infection postviral cDNA synthesis through interactions with MX2 and the HIV-1 core protein.

Previous investigations have substantially advanced our understanding of the antiviral mechanisms exhibited by SAMHD1. Biochemical experiments have demonstrated that SAMHD1 possesses dNTPase activity, and the dNTPase active site on SAMHD1 molecules is crucial for its antiviral capability (1, 27). This widely accepted model posits that SAMHD1 inhibits viral cDNA synthesis by decreasing the cellular dNTPs needed for DNA synthesis. Recent studies, however, have indicated that certain SAMHD1 mutants exhibit dNTPase activity similar to that of wild-type SAMHD1 but lose their ability to resist HIV-1 viral infection, suggesting that decreasing dNTP alone may not fully mediate the antiviral function of SAMHD1 (40, 41). Furthermore, SAMHD1 has been shown to have RNAase activity, which has been implicated in direct viral RNA degradation. Nonetheless, we confirmed the effectiveness of SAMHD1 in inhibiting HIV-1 cDNA synthesis (42). A unique aspect of our study is the discovery that SAMHD1 exerts an additional inhibitory effect beyond cDNA synthesis, preventing the nuclear entry of viral cDNA and the formation of viral 2-LTR DNA.

The identification of MX2 as an inducible antiviral restriction factor has substantially advanced our understanding of IFN-mediated inhibition of HIV-1 replication (13, 14). In addition to its antiviral activity against HIV-1, MX2 also exhibits broad inhibitory effects against herpes viruses and HBV (18, 19). Another myxovirus resistance protein, human MX1 has a broad spectrum of antiviral activities against various viruses such as influenza virus (16, 43), Semliki Forest virus (SFV) (44), classical swine fever virus (CSFV) (45), and HBV (17). In our study, we discovered that two distinct antiviral factors, SAMHD1 and MX2, can interact to form a complex that synergistically exerts antiviral effects. We also found that HIV-1 viruses with MX2-resistant capsid mutants were incapable of evading the SAMHD1-MX2-mediated synergistic antiviral effect, suggesting that the mechanisms of the SAMHD1-MX2 axis differ from their antiviral actions. Importantly, we found that the SAMHD1 proteins in different primate species can bind to MX2, indicating the evolutionary conservation of this interaction. A recent report indicated that the MX2 expression in THP-1 cells is closely associated with the antiviral function of SAMHD1 (46). These findings provide further evidence for the intricate and interwoven antiviral defense network in host cells. Diverse antiviral factors recognize and inhibit virus replication through different pathways, and these factors also exhibit synergistic interactions, resulting in novel antiviral mechanisms and increasing the challenge for viruses to evade host immune defenses. Notably, our study was primarily conducted in HIV-infected immortalized cell culture systems. Future research should use animal infection models or other *in vivo* research systems to further validate the results, which is essential for the subsequent development of targeted treatment strategies.

The influenza virus is the reference pathogen for assessing the antiviral activity of MX proteins (15, 47, 48). Human MX1 recognizes the incoming vRNPs, forming MX1 rings around these vRNPs to stall viral replication (49, 50). Similarly, MX2 inhibits HIV-1 cDNA nuclear entry by positioning near the perinuclear region of the cytoplasm and binding to the HIV-1 core (13, 14). In our study, we discovered that SAMHD1 acts as a sensor for the HIV-1 core in host cells by recognizing the HIV-1 gag component and binding to the HIV-1 core structure. Consequently, SAMHD1 can transfer the HIV-1 core protein to MX2, inhibiting the nuclear entry of viral cDNA even after its synthesis. Based on our findings, we propose a novel antiviral model in which MX2 forms a large ring-shaped complex in host cells, acting as a viral trap. In addition to directly binding viral components, MX2 can also receive viral components via other cellular cofactor interactions, delivering them to the MX2 antiviral center (Fig. 9). Further research is necessary to identify other cofactors in host cells with SAMHD1 protein-like functions that can bind viruses and transfer them to MX2, including MX1, thus tethering viruses. The development of small molecules or peptide drugs capable of recognizing viral particles, binding to MX1 or MX2, and exhibiting broad-spectrum antiviral activity is a promising avenue for future research.

In conclusion, our study revealed an additional potent antiviral effect of the SAMHD1 protein, updating the prevailing hypothesis that SAMHD1 primarily inhibits viral cDNA synthesis by regulating dNTPs levels. Notably, SAMHD1 directly targets HIV-1 viral components and synergistically influences another antiviral factor, MX2, to impede the nuclear entry of HIV-1 cDNA. Strategies aimed at increasing SAMHD1/MX2 axis activity may lead to novel therapeutics against HIV-1.

## MATERIALS AND METHODS

### Plasmid construction

SAMHD1 coding sequences were amplified by reverse transcription and PCR using mRNA samples from peripheral blood mononuclear cells of healthy donors, with the following primers: forward 5′ GTCGACACCATGCAGCGAGCCGAT 3′, reverse 5′ TCTAGATCAGGCGTAATCTGGAACATCGTATGGGTACATTGGGTCATCTTTAA 3′ containing the Sal I and Xba I sites. The PCR product was cloned and inserted into pVR1012 to generate pVR1012-SAMHD1-HA. The lentiviral expression vector pCDH-SAMHD1 and the control plasmid were gifts from Dr. Klaus Strebel (National Institutes of Health, NIH). The HIV-1-based expression vector pSCRPSY-MX2 was a generous gift from Dr. Paul D. Bieniasz (Rockefeller University). The MX2 expression plasmid Retro-X-MX2 was a generous gift from Dr. Chen Liang (McGill University). MX2 coding sequences were amplified by PCR and inserted into the pmCherry-C1 vector with Xho I and Xba I to generate pmCherry-C1-MX2.

The HIV-1 GFP reporter vector (pNL4-3-ΔEnv-EGFP, Cat# 11100) provided by Dr. Robert Siliciano (Johns Hopkins University) was obtained from the NIH AIDS Research and Reference Reagent Program, NIH. Vpx+ VLP (pSIV3+ΔEnv) and Vpx− VLP (pSIV3+ΔEnv ΔVpx) were gifts from Dr. Jacek Skowronski (Case Western Reserve University). HIV-1NL4-3 ΔEnv-GFP (wild type, G89V CA mutant), and HIV-1 GFP (wild type, Q63A/Q67A, N74D, T107N, K203A CA mutant) were obtained from Drs. Paul D. Bieniasz (Rockefeller University) and Christopher Aiken (Vanderbilt University School of Medicine).

### Cells

HEK293T cells (AIDS Research and Reference Reagents Program, Cat #3522) were maintained in Dulbecco’s modified Eagle’s medium (DMEM; Life Technologies) supplemented with 10% fetal bovine serum (FBS) and penicillin/streptomycin. The suspension cell lines U937 and K562 (ATCC) were obtained from Dr. Donald Small (Johns Hopkins University) and cultured in RPMI medium supplemented with 10% FBS. U937 cells were differentiated overnight with 30  ng/mL PMA (Promega). The HB-2 dendritic cell line was a gift from Dr. Kirk E. Sperber (The Mount Sinai Hospital) and cultured in RPMI medium supplemented with 10% FBS. Puromycin-selected cell lines were cultured in appropriate media supplemented with 1  µg mL^−1^ puromycin. shRNA-silenced cell lines were generated according to the manufacturer’s instructions (Open Biosystems) and selected for resistance to puromycin. All cultured cell lines were maintained at 37°C in a humid atmosphere containing 5% CO_2_.

### Transfection, coimmunoprecipitation, and immunoblotting

DNA transfection was carried out using Lipofectamine 2000 (Invitrogen) according to the manufacturer’s instructions. HEK293T cells were harvested at 48 h after transfection, washed twice with cold PBS, lysed in lysis buffer (150 mM Tris, pH 7.5, with 150 mM NaCl, 1% Triton X-100, and complete protease inhibitor cocktail tablets [Roche]) at 4°C for 30 min, and then centrifuged at 10,000 × *g* for 30 min. For hemagglutinin (HA) tag immunoprecipitation, precleared cell lysates were mixed with anti-HA antibody-conjugated agarose beads (Roche, catalog number 190-119) and incubated at 4°C for 3 h or overnight. The samples were then washed six times with washing buffer (20 mM Tris, pH 7.5, 100 mM NaCl, 0.1 mM EDTA, and 0.05% Tween 20). The beads were eluted with elution buffer (0.1 M glycine-HCl, pH 2.0). The eluted materials were then analyzed by SDS-PAGE and immunoblotting with the appropriate antibodies as previously described. The following antibodies were used: anti-SAMHD1 (Abcam, ab67821), anti-MX2 (Novus Biologicals, NBP1-81018), anti-cyclophilin A (Santa Cruz Biotechnology, sc-134310), anti-HA (Covance, MMS-101R-1000), anti-myc (Upstate, 05-724), anti-β-actin MAb (Sigma, A3853), and anti-GAPDH (GenScript, A00084). The monoclonal antibody against CAp24 (Cat #1513) was obtained from the AIDS Research and Reference Reagents Program.

### Identification of SAMHD1-binding proteins

HEK293T cells were transfected with pSAMHD1-HA or empty vector. After 48 h, the transfected HEK293T cells and PMA-treated U937 cells were lysed together, and the SAMHD1-containing complexes were purified from the transfected cells by immunoprecipitation using an anti-HA affinity matrix (Roche) and analyzed by SDS–PAGE. Protein samples were digested with trypsin. The peptides were separated through a Dionex Ultimate 3000 RELC nanosystem (Thermo Scientific) with a 75 µm × 15 cm Acclaim PepMap100 separating column (Thermo Scientific) protected by a 2 cm guard column (Thermo Scientific). The mobile phase flow rate was 300 nL/min with 0.1% formic acid in water (A) and 0.1% formic acid in 95% acetonitrile (B). The gradient profile was set as follows: 4%–35% B for 70 min, 35%–95% B for 5 min, 95% B for 10 min, and then equilibration in 4% B for 15 min. Mass spectrometry analysis was performed using an Orbitrap Velos Pro mass spectrometer (Thermo Scientific). The spray voltage was set at 2.2 kV. Orbitrap spectra (AGC 1 × 10^6^) were collected from 400 to 1,800 *m*/*z* at a resolution of 60 K, followed by data-dependent HCD MS/MS analysis (at a resolution of 7,500, collision energy 45%, activation time 0.1 ms) of the 10 most abundant ions using an isolation width of 2.0 Da. Charge state screening was enabled to reject unassigned and singly charged ions. A dynamic exclusion time of 35 s was used to discriminate against previously selected ions. Protein identification and label-free quantitation were performed using MaxQuant ver. 1.3.0.5, which was searched against a human protein database version 3.87, containing a total of 91,464 entries. Default parameters with a 1% false-discovery rate (FDR) for MaxQuant were used, except the following parameters: enzyme, trypsin; filter-labeled amino acid, unselected; and match between runs, 2 min. Label-free quantification (LFQ) and iBAQ features were selected.

### Viruses

For HIV-1 GFP production, HEK293T cells were transfected with 9  µg of HIV-1ΔENV-GFP and 3  µg of pCMV-VSV-G encoding plasmid; for shRNA production, HEK293T cells were transfected with 5  µg of shRNA construct, 5  µg of packaging plasmid pΔ8.91, and 2  µg of VSV-G encoding plasmid; and for Vpx+ VLP or Vpx− VLP production, 9  µg of pSIV3 + ΔEnv or pSIV3 + ΔEnv ΔVpx was cotransfected with or without 9  µg of VSV-G-encoding plasmid. The medium was replaced 12 h after transfection, and the viruses were harvested 48 h later, and then filtered at 0.45 µm. The supernatants were then purified, and the lentivirus was concentrated by ultracentrifugation at 28 K for 1.5 h with a 20% sucrose cushion. The virus pellet was then dissolved in PBS and stored at −70°C.

For single infection with HIV-1-GFP virus, U937, K562, or 293T cells were plated in 24-well plates. In some experiments, cells were treated with Vpx+ VLP or Vpx− VLP 2 h prior to HIV-1-GFP infection. The efficiency of productive infection was analyzed 48 h later using flow cytometry (FACSCalibur, BD Biosciences).

### RNA interference

shRNA DNA clone that targets MX2 (shMX2, TRCN0000056713) was a gift from Dr. Chen Liang (McGill University) (15). shMX2 or the control vector was produced by cotransfection of HEK293T cells with pΔ8.91 and the pCMV-VSVG vector, and U937 cells were transduced with filtered supernatant overnight before medium replacement. Stably transduced cells were selected with puromycin (0.5 µg/mL). SAMHD1 was transiently expressed in control or MX2-silenced U937 cells using CDH-SAMHD1 or CDH viruses. Two days later, HIV-1 GFP viruses were used to infect various transduced U937 cells, various transduced U937 cells were infected with the HIV-1 GFP virus, and the number of GFP-positive cells was determined by flow cytometry 48 h after infection.

For siRNA-mediated MX2 silencing, HB-2 cells were transfected with siRNA oligonucleotides against MX2 or with no-target control siRNA (ON-TARGETplus smart pool; Dharmacon) using Lipofectamine 2000 according to the manufacturer’s instructions. At 18 h before infection with the HIV-1 virus, the transfected cells were treated with IFN-α (500  U/mL, PBL Interferon Source).

### Step gradient analyses

For step sucrose gradient analysis, the purified virus was dissolved in PBS buffer, briefly exposed to 0.1% Triton X-100, and loaded onto a step sucrose gradient as previously described. Three fractions were harvested: one containing soluble proteins (F1), one containing a buffer fraction (F2), and one containing a virus core-containing fraction (F3). The individual gradient fractions were then subjected to immunoblot analysis.

### Quantitative real-time PCR

Cells were collected at 12 h post-infection and washed with PBS, and total DNA was extracted using the QIAamp DNA Blood Mini Kit (Qiagen). The DNA samples were analyzed by quantitative PCR using FastStart Universal SYBR Green Master Mix (Roche) and an ABI 7000 sequence detection system (Applied Biosystems). The primers for the detection of early reverse transcription (ERT), late reverse transcription (LRT), and 2-LTR circular DNA were as follows: ERT, 5′ TTAGACCAGATCTGAGCCTGGGAG 3′ and 5′ GGGTCTGAGGGATCTCTAGTTACC 3′; LRT, 5′ TGTGTGCCCGTCTGTTGTG 3′ and 5′ GAGTCCTGCGTCGAGA 3′; and 2-LTR, 5′ CCCTCAGACCCTTTTAGTCAGTG 3′ and 5′ TGGTGTGTAGTTCTGCCAATCA 3′.

### Statistical analyses

The statistical data were analyzed using GraphPad Prism software (version 8.0; GraphPad Software Inc., San Diego, CA, USA). Differences among test groups were analyzed by ANOVA. A value of *P* < 0.05 was considered to indicate statistical significance.

## Data Availability

The data that support the findings of this study are available from the corresponding author upon reasonable request.

## SUPPLEMENTAL MATERIAL

Supplemental Figures mbio.01363-24-s0001.pdf Figures S1 to S3.

ASM does not own the copyrights to Supplemental Material that may be linked to, or accessed through, an article. The authors have granted ASM a non-exclusive, world-wide license to publish the Supplemental Material files. Please contact the corresponding author directly for reuse.
