# Supplementary material for: The SAMHD1-MX2 axis restricts HIV-1 infection at postviral DNA synthesis
Source: mBio. 2024 Jun 18;15(7):e01363-24. doi: 10.1128/mbio.01363-24 (PMC11253599; doi:10.1128/mbio.01363-24)
Supplement: Supplemental Figures — Figures S1 to S3. [file mbio.01363-24-s0001.pdf]

Supplementary Information

The SAMHD1-MX2 Axis Restricts HIV-1 Infection at Postviral DNA Synthesis

Guo et. al

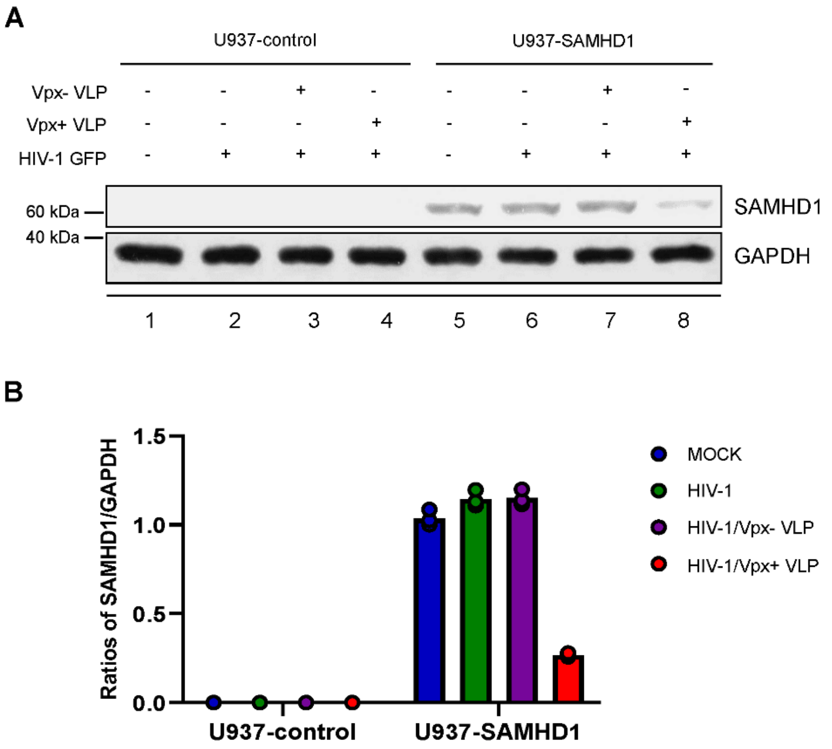

Figure S1. Vpx triggers SAMHD1 degradation in SAMHD1-U937 cells. A) PMA-stimulated control or SAMHD1-expressing U937 cells were infected with (lanes 2, 6) or without (lanes 1, 5) the HIV-1 GFP virus in the presence of Vpx+ VLP (lanes 4, 8) or Vpx- VLP (lanes 3, 7). Twelve hours later, the cells were harvested for immunoblot analysis, and the indicated antibodies were used to detect SAMHD1 and the loading control protein GAPDH. Vpx+ VLP induced SAMHD1 degradation (lane 8). B) Quantified relative expression levels of SAMHD1.

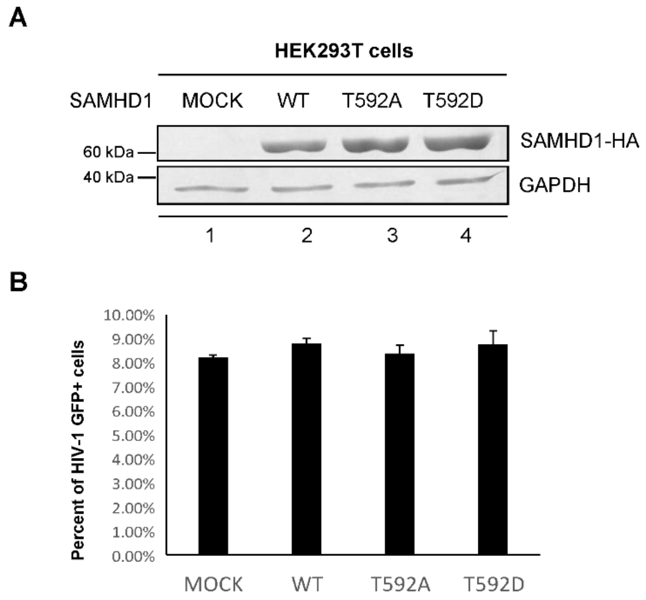

Figure S2. SAMHD1 expression does not affect HIV-1 infection in HEK293T cells. A) Immunoblot analysis of SAMHD1 expression in pSAMHD1-HA wild-type (WT), T592A-, and T592D-transfected HEK293T cells. B) Transfected HEK293T cells were infected with equivalent amounts of HIV-1-GFP virus. The cells were harvested, and the percentage of HIV-1 GFP-positive cells was determined by flow cytometry 2 days after infection.

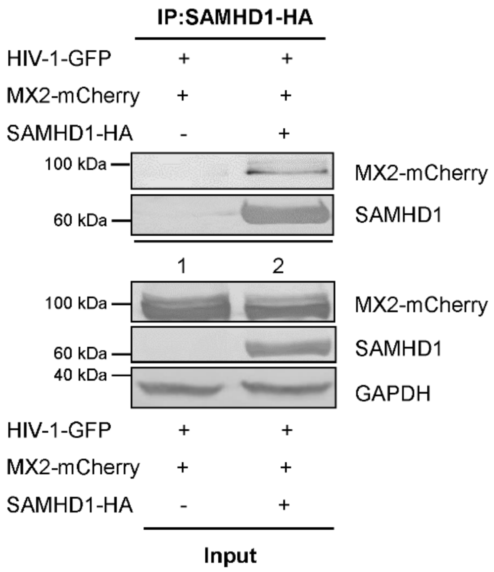

Figure S3. Characterization of the interaction between SAMHD1 and MX2 in the presence of HIV-1 infection. Co-IP of MX2-mCherry with SAMHD1-HA from HIV-1-GFP pseudovirus-infected HEK293T cell samples.
